# Development and maintenance of a medical education research registry

**DOI:** 10.1186/s12909-020-02113-5

**Published:** 2020-06-19

**Authors:** Jeffrey A. Wilhite, Lisa Altshuler, Sondra Zabar, Colleen Gillespie, Adina Kalet

**Affiliations:** 1 Department of Medicine, Division of General Internal Medicine and Clinical Innovation, NYU Robert I. Grossman School of Medicine, 462 1st Avenue, New York, NY 10016 USA; 2 Institute for Innovations in Medical Education, Division of Education Quality, 550 First Avenue, Medical Science Building, Suite G107, New York, NY 10016 USA; 3 Robert D. and Patricia E. Kern Institute for the Transformation of Medical Education, Medical College of Wisconsin, 8701 W. Watertown Plank Road, Wauwatosa, WI 53226 USA

**Keywords:** Medical education, Research registry, Graduate medical education, Undergraduate medical education, Medical education research

## Abstract

**Background:**

Medical Education research suffers from several methodological limitations including too many single institution, small sample-sized studies, limited access to quality data, and insufficient institutional support. Increasing calls for medical education outcome data and quality improvement research have highlighted a critical need for uniformly clean and easily accessible data. Research registries may fill this gap. In 2006, the Research on Medical Education Outcomes (ROMEO) unit of the Program for Medical Innovations and Research (PrMEIR) at New York University’s (NYU) Robert I. Grossman School of Medicine established the Database for Research on Academic Medicine (DREAM). DREAM is a database of routinely collected, de-identified undergraduate (UME, medical school leading up to the Medical Doctor degree) and graduate medical education (GME, residency also known as post graduate education leading to eligibility for specialty board certification) outcomes data available, through application, to researchers. Learners are added to our database through annual consent sessions conducted at the start of educational training. Based on experience, we describe our methods in creating and maintaining DREAM to serve as a guide for institutions looking to build a new or scale up their medical education registry.

**Results:**

At present, our UME and GME registries have consent rates of 90% (*n* = 1438/1598) and 76% (*n* = 1988/2627), respectively, with a combined rate of 81% (*n* = 3426/4225). 7% (*n* = 250/3426) of these learners completed both medical school and residency at our institution. DREAM has yielded a total of 61 individual studies conducted by medical education researchers and a total of 45 academic journal publications.

**Conclusion:**

We have built a community of practice through the building of DREAM and hope, by persisting in this work the full potential of this tool and the community will be realized. While researchers with access to the registry have focused primarily on curricular/ program evaluation, learner competency assessment, and measure validation, we hope to expand the output of the registry to include patient outcomes by linking learner educational and clinical performance across the UME-GME continuum and into independent practice. Future publications will reflect our efforts in reaching this goal and will highlight the long-term impact of our collaborative work.

## Background

Medical education research (MER) should and could improve the health of the public by informing policy and practice but it suffers from many methodological limitations including small sample sizes, cross sectional designs, and lack of attention to important context variables. There are increasing calls for medical education research, a poorly funded field, to go beyond the proximate outcomes of training to study more distal clinical outcomes using “big data” strategies [1, 2]. And yet, even within the same institution, data is collected using different systems and a wide range of formats, without a shared ontology or structured language and therefore is not organized to enable longitudinal tracking of learners, learning, or linking with outcomes.

While medical trainees must be afforded the same ethical and legal protections as any research subjects and U.S. federal regulations allow the use of educational data collected in the routine conduct of a required curriculum for research without written consent from learners, medical school research ethics review boards are not consistent in their approach to trainees as study subjects, complicating the ethical conduct of this type of research [3]. Establishing research registries can help overcome some of these barriers and facilitate higher impact, ethically rigorous programs of medical education research [4].

Research registries compile and maintain multiple-source, standardized information on potential study participants longitudinally for many purposes [5]. The National Leprosy Registry in Norway established in 1856, was the earliest disease specific registry, and the number of disease specific registries has increased steadily since [6]. More recently, research registries have focused on data integrity and quality improvement, following in the footprints of the Framingham Heart Study-style that began in 1970 [5, 7]. In 2006, the Research on Education Outcomes (ROMEO) unit of the Program for Medical Innovations and Research (PrMEIR) at New York University’s (NYU) School of Medicine established the Database for Research on Academic Medicine (DREAM) with funding from the Bureau of Health Professions (BHPr) of the department of Human Resource Services Administration (HRSA # D54HP05446). Borrowing from the constructs underlying disease registries, the goal of DREAM is to enable ethical, longitudinal study of outcomes in medical education through the collection of routine trainee and educational context data [8]. DREAM, a potential database assembled as needed to ask and answer specific research questions, is structured as two research registries - one for Medical Students (established in 2008, with 1438 current individuals enrolled) and the second, a Graduate Medical Education Registry (established 2006, with 1988 current individuals enrolled), for trainees in the twenty participating residency and fellowship programs in our institution.

With approval from NYU’s Institutional Review Board (IRB) for our medical student and resident/fellow registries, we have been collecting written informed consent from medical trainees (students, residents, and fellows) using highly structured, transparent procedures. We request permission to compile data collected as a routine, required part of the trainee’s education for use in medical education research. Consenting subjects are informed both verbally and in writing that only data that has been de-identified by DREAM’s Honest Broker- a specific individual is responsible for serving as the data steward for the registries and who has absolutely no involvement in the selection, recruitment, employment, evaluation or education of the potential subjects- will be made available for the purposes of research. The consenting process is conducted by core members of the research team serving as Honest Brokers to ensure that the students’ and residents’ decision to consent is private and will not have repercussions (perceived or actual) for their standing in medical school.

DREAM is a collection of routinely gathered educational data spanning the undergraduate medical education (UME, medical school leading up to the Medical Doctor degree)-graduate medical education (GME, residency training also known as post graduate education leading to eligibility for specialty board certification) continuum. Available data at the UME level includes admissions data (e.g., Medical College Admission Test (MCAT) scores, Bachelor’s Degree Grade Point Average (GPA) and field of study (also referred to as major), medical school admissions multiple-mini interview scores), academic performance data (e.g., medical knowledge exam scores, clinical clerkship grades, clinical and workplace-based assessments), performance-based assessments (e.g., Objective Structured Clinical Examinations (OSCEs) and simulations), national licensing exams (Specialty Shelf, Step 1 and Step 2 Exams), and assessments of professional development. Available data at the GME level depends on the participating residency program but generally includes admissions data (medical school, Step 1 and Step 2 Clinical Knowledge (CK) exam scores), attitude and clinical experience/training surveys, milestone assessments, performance-based assessments (OSCEs and simulations), national exams (specialty certification board scores) and clinical assessments (e.g., workplace-based assessments, clinical and procedural skill assessments, and Unannounced Standardized Patient visits). We are currently engaged in several initiatives to incorporate clinical process and outcome data, at least partly attributable to residents, including primary care panel process and outcome variables, laboratory test ordering, and relevant practice quality metrics. As part of the GME Registry, we ask permission to join UME and GME data of learners who graduated from both levels at NYU School of Medicine, and currently maintain a database of 250 continuum learners.

**Fig. 1:**
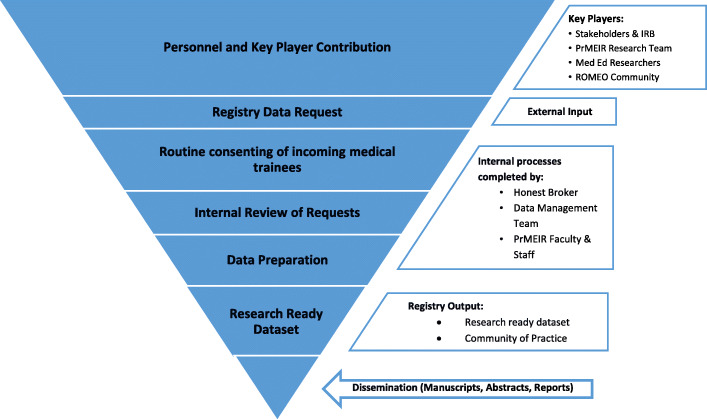
Registry Component Funnel

**Fig. 2:**
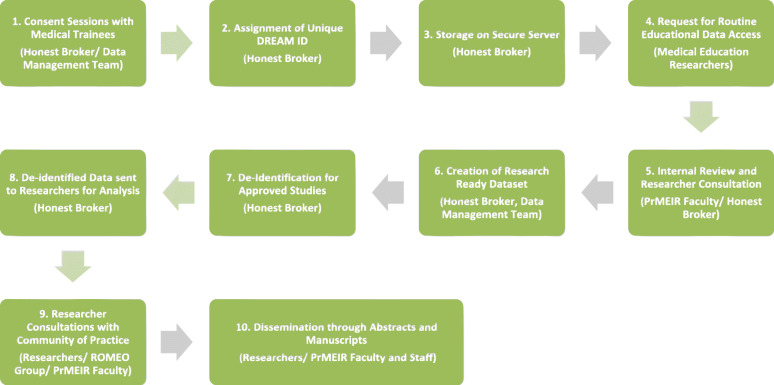
The Research Registry Roadmap (Responsible Party)

As far as we know the DREAM registries are the longest standing comprehensive medical education registries globally. To date 61 studies have been approved that utilize DREAM data (Table 1). Of these studies, there have been 45 publications, answering research questions focused on evaluation of trainings or curriculum, assessment of learner core competencies, and/ or validation of measures (Table 1, Table 2). Routine Registry processes and flow have developed longitudinally through continual internal review and improvement (Fig. 1, Fig. 2). Based on this accumulated experience and scholarly productivity, we propose 3 overarching principles and related practical advice to the larger medical education community on how to develop, implement, maintain and maximize the productivity of a MER registry. While our registries, and the data privacy issue examples provided are specific to the United States context, we suspect that the underlying goals, objectives, principles and guidance provided can be applied in general to the international MER community.

**Table 1:** Approved Studies, Subsequent Publications, and Publication Core Research Question Areas

**Registry**	**# Approved Studies to Date**	**# with Publications**	**Total # of Publications**
**UME (Medical Student)**	**29**	**15**	**17**
**GME (Resident/Fellow)**	**32**	**15**	**28**
**TOTAL**	**61**	**30**	**45**
**Core Research Question Area (Non-Exclusive)**		**Number of Publications by Core Research Question**	
**Assessment of Learner Core Competencies**		**22**	
**Evaluation of Training Programs or Curriculum**		**23**	
**Validity of Measures**		**4**

**Table 2:** Listing of Registry-Related Publications

UME or GME Registry	Citation
**GME**	**Altshuler L, Fisher H, Hanley K, Ross J, Zabar S, Adams J, Lipkin M. Training Primary Care Physicians to Serve Underserved Communities: Follow-up Survey of Primary Care Graduates. Journal of general internal medicine. 2019 Dec 1;34(12):2728–30.**
**GME**	**Boutis K, Pecaric M, Carrière B, Stimec J, Willan A, Chan J, Pusic M. The effect of testing and feedback on the forgetting curves for radiograph interpretation skills. Medical teacher. 2019 Apr 29:1–9.**
**UME**	**Crotty, K; Robinson, A; Gillespie, C; Schaye, V; Grogan, K; Tewksbury, L. Md aware: Qualitatively measuring the impact of longitudinal resiliency curriculum and wellbeing self-assessment tool among medical students. Journal of general internal medicine. 2019:Conference:(2019).**
**UME**	**Fang V, Gillespie C, Crowe R, Popeo D, Jay M. Associations between medical students’ beliefs about obesity and clinical counseling proficiency. BMC obesity. 2019 Dec;6(1):5.**
**UME**	**Hatala R, Gutman J, Lineberry M, Triola M, Pusic M. How well is each learner learning? Validity investigation of a learning curve-based assessment approach for ECG interpretation. Advances in Health Sciences Education. 2019 Mar 1;24(1):45–63.**
**GME**	**Lee MS, Pusic M, Carrière B, Dixon A, Stimec J, Boutis K. Building emergency medicine trainee competency in pediatric musculoskeletal radiograph interpretation: a multicenter prospective cohort study. AEM education and training. 2019 Jul;3(3):269–79.**
**GME**	**Wilhite JA, Velcani F, Watsula-Morley A, Hanley K, Altshuler L, Kalet A, Zabar S, Gillespie CC. Igniting activation: Using unannounced standardized patients to measure patient activation in smoking cessation. Addictive behaviors reports. 2019 Jun 1;9:100179.**
**GME**	**Zabar S, Hanley K, Horlick M, Cocks P, Altshuler L, Watsula-Morley A, Berman R, Hochberg M, Phillips D, Kalet A, Gillespie C. “I Cannot Take This Any More!”: Preparing Interns to Identify and Help a Struggling Colleague. Journal of general internal medicine. 2019 May 15;34(5):773–7.**
**GME**	**Crotty KJ, Felson S, Leung J, Felson J. Evaluating an innovative VA resident group practice model in block scheduling. Journal of general internal medicine. 2018;Conference:(41st):184–185.**
**UME**	**Crotty, K J; Robinson, A; Grogan, K; Schaye, V; Gillespie, C; Tewksbury, L. Measuring the impact of longitudinal resiliency curriculum and wellbeing self-assessment tool among medical students. Journal of general internal medicine. 2018:Conference:(41st).**
**UME**	**Eliasz KL, Ark TK, Nick MW, Ng GM, Zabar S, Kalet AL. Capturing Entrustment: Using an End-of-Training Simulated Workplace to Assess the Entrustment of Near-graduating Medical Students from Multiple Perspectives. Medical Science Educator. 2018 Dec 15;28(4):739–47.**
**UME**	**Kalet A, Buckvar-Keltz L, Monson V, Harnik V, Hubbard S, Crowe R, Ark TK, Song HS, Tewksbury L, Yingling S. Professional Identity Formation in medical school: One measure reflects changes during pre-clerkship training. MedEdPublish. 2018 Feb 21;7.**
**UME**	**Lewis A, Howard J, Watsula-Morley A, Gillespie C. An educational initiative to improve medical student awareness about brain death. Clinical neurology and neurosurgery. 2018 Apr 1;167:99–105.**
**GME**	**Rostanski SK, Kurzweil AM, Zabar S, Balcer LJ, Ishida K, Galetta SL, Lewis A. Education Research: Simulation training for neurology residents on acquiring tPA consent: An educational initiative. Neurology. 2018 Dec 11;91(24):e2276–9.**
**GME**	**Zabar S, Hanley K, Watsula-Morley A, Goldstein J, Altshuler L, Dumorne H, Wallach A, Porter B, Kalet A, Gillespie C. Using Unannounced Standardized Patients to Explore Variation in Care for Patients With Depression. Journal of graduate medical education. 2018 Jun;10(3):285–91.**
**GME**	**Greene RE, Hanley K, Cook TE, Gillespie C, Zabar S. Meeting the primary care needs of transgender patients through simulation. Journal of graduate medical education. 2017 Jun;9(3):380–1.**
**GME**	**Hanley K, Zabar S, Altshuler L, Lee H, Ross J, Rivera N, Marvilli C, Gillespie C. Opioid vs nonopioid prescribers: Variations in care for a standardized acute back pain case. Substance abuse. 2017 Jul 3;38(3):324–9.**
**UME**	**Kalet A, Buckvar-Keltz L, Harnik V, Monson V, Hubbard S, Crowe R, Song HS, Yingling S. Measuring professional identity formation early in medical school. Medical Teacher. 2017;39:3255–261.**
**UME**	**Kalet A, Zabar S, Szyld D, Yavner SD, Song H, Nick MW, Ng G, Pusic MV, Denicola C, Blum C, Eliasz KL. A simulated “Night-onCall” to assess and address the readiness-for-internship of transitioning medical students. Advances in Simulation. 2017 Dec;2(1):13.**
**GME**	**Altshuler L, Plaksin J, Zabar S, Wallach A, Sawicki C, Kundrod S, Kalet A. Transforming the patient role to achieve better outcomes through a patient empowerment program: A randomized wait-list control trial protocol. JMIR research protocols. 2016;5(2):e68.**
**UME**	**Gershgorin I, Marin M, Xu J, Oh SY, Zabar S, Crowe R, Tewksbury L, Ogilvie J, Gillespie C, Cantor M, Aphinyanaphongs Y, Kalet A. Using natural language processing to automate grading of students’ patient notes: Proof of concept. Journal of general internal medicine. 2016;31:S458-S458.**
**GME**	**Hochberg MS, Berman RS, Kalet AL, Zabar S, Gillespie C, Pachter HL. Professionalism training for surgical residents: documenting the advantages of a professionalism curriculum. Annals of surgery. 2016 Sep 1;264(3):501–7.**
**GME**	**Winkel AF, Gillespie C, Uquillas K, Zabar S, Szyld D. Assessment of developmental progress using an objective structured clinical examination-simulation hybrid examination for obstetrics and gynecology residents. Journal of surgical education. 2016 Mar 1;73(2):230–7.**
**GME**	**Zabar S, Adams J, Kurland S, Shaker-Brown A, Porter B, Horlick M, Hanley K, Altshuler L, Kalet A, Gillespie C. Charting a key competency domain: understanding resident physician interprofessional collaboration (IPC) skills. Journal of general internal medicine. 2016 Aug 1;31(8):846–53.**
**UME**	**Paul S, Pusic M, Gillespie C. Medical student lecture attendance versus iTunes U. Medical education. 2015 May;49(5):530–1.**
**UME**	**Bhatia ND, Gillespie CC, Berger AJ, Hochberg MS, Ogilvie JB. Cutting too deep? Assessing the impact of a shorter surgery clerkship on students’ clinical skills and knowledge. The American Journal of Surgery. 2014 Feb 1;207(2):209–12.**
**UME**	**Hanley K, Zabar S, Charap J, Nicholson J, Disney L, Kalet A, Gillespie C. Self-assessment and goal-setting is associated with an improvement in interviewing skills. Medical education online. 2014 Jan 1;19(1):24407.**
**GME**	**Winkel AF, Gillespie C, Hiruma MT, Goepfert AR, Zabar S, Szyld D. Test of integrated professional skills: objective structured clinical examination/simulation hybrid assessment of obstetrics-gynecology residents’ skill integration. Journal of Graduate Medical Education. 2014 Mar;6(1):117–22.**
**GME**	**Zabar S, Gillespie C, Hanley K, Kalet A. Directly Observed Care: Can Unannounced Standardized Patients Address a Gap in Performance Measurement?. Journal of General Internal Medicine. 2014 Nov 1;29(11):1439.**
**GME**	**Zabar S, Hanley K, Stevens D, Murphy J, Burgess A, Kalet A, Gillespie C. Unannounced standardized patients: a promising method of assessing patient-centered care in your health care system. BMC health services research. 2014 Dec;14(1):157.**
**GME**	**Hochberg MS, Berman RS, Kalet AL, Zabar SR, Gillespie C, Pachter HL. The stress of residency: recognizing the signs of depression and suicide in you and your fellow residents. The American Journal of Surgery. 2013 Feb 1;205(2):141–6.**
**GME**	**Jay MR, Gillespie CC, Schlair SL, Savarimuthu SM, Sherman SE, Zabar SR, Kalet AL. The impact of primary care resident physician training on patient weight loss at 12 months. Obesity. 2013 Jan;21(1):45–50.**
**UME**	**Pusic MV, Gillespie C. On showing all the ripples in the growth analysis pond. Medical education. 2013 Jul;47(7):643–5.**
**UME**	**Berger AJ, Gillespie CC, Tewksbury LR, Overstreet IM, Tsai MC, Kalet AL, Ogilvie JB. Assessment of medical student clinical reasoning by “lay” vs physician raters: inter-rater reliability using a scoring guide in a multidisciplinary objective structured clinical examination. The American Journal of Surgery. 2012 Jan 1;203(1):81–6.**
**GME**	**Hochberg MS, Berman RS, Kalet AL, Zabar SR, Gillespie C, Pachter HL, Interpersonal Communications Education Study Group. The professionalism curriculum as a cultural change agent in surgical residency education. The American Journal of Surgery. 2012 Jan 1;203(1):14–20.**
**GME**	**Pusic MV, Andrews JS, Kessler DO, Teng DC, Pecaric MR, Ruzal-Shapiro C, Boutis K. Prevalence of abnormal cases in an image bank affects the learning of radiograph interpretation. Medical education. 2012 Mar;46(3):289–98.**
**GME**	**Pusic MV, Kessler D, Szyld D, Kalet A, Pecaric M, Boutis K. Experience curves as an organizing framework for deliberate practice in emergency medicine learning. Academic Emergency Medicine. 2012 Dec;19(12):1476–80.**
**GME**	**Pusic MV, Pecaric M, Boutis K. How much practice is enough? Using learning curves to assess the deliberate practice of radiograph interpretation. Academic Medicine. 2011 Jun 1;86(6):731–6.**
**GME**	**Hochberg MS, Kalet A, Zabar S, Kachur E, Gillespie C, Berman RS. Can professionalism be taught? Encouraging evidence. The American Journal of Surgery. 2010 Jan 1;199(1):86–93.**
**GME**	**Jay M, Gillespie C, Schlair S, Sherman S, Kalet A. Physicians’ use of the 5As in counseling obese patients: is the quality of counseling associated with patients’ motivation and intention to lose weight?. BMC health services research. 2010 Dec;10(1):159.**
**GME**	**Jay M, Schlair S, Caldwell R, Kalet A, Sherman S, Gillespie C. From the patient’s perspective: the impact of training on resident physician’s obesity counseling. Journal of general internal medicine. 2010 May 1;25(5):415–22.**
**GME**	**Kramer V, Friedenberg AS, Bonura E, Gillespie C, Smith R, Kaufman B, Felner K. Development and application of a behavior-based tool to assess internal medicine resident leadership skills using a high-fidelity patient simulator. Decision-Making, 2010, 34, 28–4.**
**GME**	**Gillespie C, Paik S, Ark T, Zabar S, Kalet A. Residents’ perceptions of their own professionalism and the professionalism of their learning environment. Journal of graduate medical education. 2009 Dec;1(2):208–15.**
**UME**	**Stevens DL, King D, Laponis R, Hanley K, Zabar S, Kalet AL, Gillespie C. Medical students retain pain assessment and management skills long after an experiential curriculum: a controlled study. Pain. 2009 Oct 1;145(3):319–24.**
**GME**	**Zabar S, Ark T, Gillespie C, Hsieh A, Kalet A, Kachur E, Manko J, Regan L. Can unannounced standardized patients assess professionalism and communication skills in the emergency department?. Academic Emergency Medicine. 2009 Sep;16(9):915–8.**

## Principle 1: ensure the MER registry is addressing an important research mission

### Invite broad input from stakeholders

Assembling a broadly representative team of key stakeholders early in the project ensures long-term buy-in and maximizes resource support and registry productivity [9]. During initial planning phases, our discussions with community members, including medical education researchers, trainees, information technology (IT) experts, and course, clerkship and residency program directors honed registry objectives and built a case for a medical education research registry at the School of Medicine. A major objective of the registry, developed in collaboration with stakeholders, is to provide clean, easily accessible, de-identified data to the medical education community at NYU while maintaining strict confidentiality and data security. These types of data sets are exceedingly rare in medical education compared to other fields [10, 11].

Stakeholder relationships continue to deepen because projects using DREAM data are regularly discussed at weekly, multi-disciplinary sessions where work in progress is shared and collaborations are nurtured. In addition, our review and approval processes include consultations and advising on available data resources, research design and methods, analytic approaches and opportunities for collaboration and therefore there is a great deal of informal MER faculty development that has helped build and sustain a community of Medical Education Researchers (Fig. 2).

### Identify the scope and purpose of the registry

It is critical to define the scope and purpose of the MER registry [5]. Given our funding and particular areas of interest, the long term goal of DREAM is to establish the evidence base to guide medical education policy, innovation and practice by directly linking medical education with care quality and safety outcomes for patients. Our registries incorporate selective demographic and descriptive data about learners (e.g. Admissions), all routinely collected assessment and performance data, and selected education-focused survey data. For residents and fellows, we collect de-identified patient data directly attributable to those clinicians (e.g. ambulatory care-based patient panel data), and research utilizing this data is in progress [8].

Our registry was developed in close collaboration with NYU’s Human Subjects Institutional Review Board (IRB), the Office of Medical Education responsible for running the medical school, and individual residency and fellowship program directors [8]. Carefully articulating the purpose of a medical education registry helps to maximize stake-holder involvement and buy-in (e.g. trainees, faculty, administrators, patients, the community, the IRB), and to avoid potential misunderstandings of the registry process [12]. While building the argument for the need for the registry, creators should identify, become conversant with and address critical issues relevant to implementation including the privacy rights and concerns of trainees and legal concerns of the educational and training programs. In the U.S. this means being familiar with both the Health Insurance Portability and Accountability Act of 1996 (HIPAA) and The Family Educational Rights and Privacy Act of 1974 (FERPA) federal laws addressing relevant privacy rights. Data for the registry is maintained by institutional entities and housed on highly secure Medical Center Information Technology (MCIT) servers. The DREAM registries protocols describe how researchers can obtain research-ready curated sets of data (fully de-identified data for only those learners that have provided consent).

Ultimately, the DREAM registries not only serve to advance medical education research globally, they also have contributed substantially to educational scholarship locally, supported promotion and career advancement for our education faculty, and helped faculty and education leaderships answer important – and almost always generalizable – questions about our local curriculum.

### Establish a vision and set goals related to future data needs

Registry creators should remain aspirational and regularly brainstorm new research questions and protocols. Our initial plans, which included a focus on collecting performance-based assessment data (e.g. mostly from OSCEs), has grown to include additional assessment data (e.g. knowledge tests, peer assessments, mid-clerkship formative feedback). We also document the nature and timing of significant admissions and curriculum changes. Through regular registry data requests, the PrMEIR team has identified and addressed barriers to filling these requests. These barriers include technical issues such as a variety of data naming, storage, format and complexity concerns, as well as differing views on data use from course and curriculum leadership, registrar, human resources and clinical administrators despite consent from trainees (e.g. “who’s data is it?”). With increasing awareness of the registry and clarification of its purpose we are seeing an increase in the number and complexity of data requests. This has pushed us to continuously improve our communication with stakeholders, clarify our written materials, and enhance our capacity for data wrangling and management [13]. For instance, we have identified a goal of creating and implementing a standardized education research data warehouse with complete sets of data by student cohort [8, 11]. Ideally this warehouse, or mart, will become a one-stop-shop for commonly requested data elements [8] that would permit aggregate data queries for planning studies and extraction of de-identified data sets for research. The Honest Broker, in more of a Data Steward role (see Principle 2 below) would help maintain the warehouse and routinely adjust its contents, structure, and data definitions to meet demands of an enlarging MER community. In our case, because financial support for DREAM staff has been provided through a combination of research and data infrastructure grants updating to a more robust and comprehensive data architecture is not yet possible. We are optimistic that as institutional data/ information technology resources become steadily more sophisticated more support will become available.

## Principle 2: utilize best practice policies and procedures in establishing a MER

### Assign a honest broker

A registry should be managed by a data steward, or an individual positioned as the Honest Broker of research data. In clinical research, Honest Brokers are HIPAA compliant data preparers with a detailed understanding of the role of privacy, confidentiality and data security as regulated by legal requirement and sound moral judgement [14]. A steward should be capable of carrying out routine data management practices and able to maintain IRB and collaborator communication [14]. Ideally, Honest Brokers serve as a neutral intermediary between researchers and research participants and in the context of medical education, are responsible for safeguarding a participant’s protected education and training data [15, 16]

PrMEIR’s Honest Broker is usually an individual with master’s level education and training specific to adhering to the rules of medical trainee privacy protection. The Broker conducts informed consent procedures with potential participants, prepares clean, de-identified data, communicates with interested collaborators, maintains a relationship with the IRB, is present during all registry related discussions, disseminates appropriate pieces of information to stakeholders, and most importantly, holds, privately and securely, the information on trainees’ decision to consent. Salary support for this function (e.g. calculated as % of a full-time equivalent (FTE)) is provided by a combination of external grants and institutional operating budget.

### Establish appropriate funding channels and designate faculty and staff time

Creation of the DREAM registries was funded primarily through three successive Academic Administrative Unit grants from the US Health Resources and Services Administration (HRSA) running from 2011 to 2017. The aim of these grants was, among other things, to build the evidence base for medical education by supporting the data infrastructure necessary for linking educational efforts to patient outcomes in primary care. During the initial days of the registry, several core faculty members spent about a year designing the UME and GME registries and working with the NYU Robert I. Grossman School of Medicine’s IRB to finalize the protocol, consent materials and standard operating procedures. During this period, a small portion of funding was used to obtain the input of a data scientist, with the remainder going toward salary support of PrMEIR staff and faculty. Data storage was, and remains supported through NYU’s MCIT. Since then, grant funding has gone directly to PrMEIR faculty and staff (Honest Broker, Data Management Team) support in the realms of consenting, data cleaning, and upkeep of the registry.

Maintaining the two registries involves annual IRB continuation applications, consenting new learners each year, logging those consents, processing requests to participate in the registry, and overseeing data management. PrMEIR’s Honest Broker (author JW) spends approximately .40 FTE on registry upkeep. Data management, including data cleaning, rationalization (variable labeling, building and maintaining data dictionaries), merging/linking of data sets, and secure data storage, requires the greatest share of resources and currently includes .50 FTE of a Data Manager and .25 FTE of a Data Analyst. When grant-funded studies use core data from the Registries, small amounts of funding have been allocated to supporting Registry Data Management. The Principal Investigator (PI), or lead PrMEIR faculty for each Registry’s IRB protocol (authors CG and SZ), ensures that protocol is followed and participates as part of a small advisory committee that reviews and approves Registry requests, advises on data management practices, and makes use of the data for education research locally. This represents an estimated .05 FTE effort for each of the PIs and for each of the three advisory committee faculty members. A PrMEIR Program Administrator provides supervision to the Honest Broker and Data Management Team (.10 FTE). Therefore, the full team represents about 1.5 total FTE annually. Upkeep of the registry has since been supported in small ways through philanthropy-based funds and portions of other grants from external sources including AHRQ and foundation funding. There was little buy-in from the institution during early development phases, but this has shifted due to the meaningful output of research from those who utilize registry data.

### Create rigorous, regulatory-compliant consent materials and processes

IRB approved consent forms (stamped with the approval date) describe the project, anticipated benefits and potential risks as well as processes for consenting, withdrawal of consent and reporting of coercion and breaches of privacy [17]. The Honest Broker (or delegated research team member) obtains trainee consents by attending new medical trainee program orientations to conduct a brief (< 30 min) information session. He or she briefly describes the project, allows time for trainees to read the consent form, answers questions, and collects signed, written consents from incoming learners. Trainees place their consent forms, whether they provided consent or not, in an envelope and all are asked to return the envelope in order to preserve the privacy and confidentiality of their decision to consent. Beforehand, the Honest Broker coordinates attendance with departmental leadership, collects rosters of incoming students for registry record keeping and prepares materials (printed consent forms, rosters, and attendance tracking materials).

The names of all trainees who consent to a registry are entered into the secure database (REDCap) within 24 h of collection from learners [18]. Each consenting learner is assigned a unique DREAM Identification number (DREAM ID). The Honest Broker maintains this “cross walk” database connecting subject names and DREAM IDs which is kept on a secure, firewall-protected server to which only a very limited set of research team members have access (currently, *n* = 2). Signed consent forms are organized consecutively and kept in a locked file cabinet. No one but the Honest Broker and core research team has access to information on the consent status of any individual trainee. Institutional collaborators are then able to request, from the PrMEIR team, curated sets of this data for research studies [8]. Collaborators must meet strict guidelines for usage. Namely, their study cannot be experimental, must be for education evaluation, assessment, or improvement purposes, and must identify each individual (Table 4).

Approximately 90% of medical students (UME) and 76% of residents and fellows (GME) consent to the DREAM registries annually (Table 3). Registry personnel complete and maintain all required research staff training. At our institution this includes the Collaborative Institutional Training Initiative (CITI Program) training on biomedical and social/ behavioral research, and good clinical practice. The PrMEIR faculty must maintain Principal Investigator Development and Resources (PINDAR) training completion certificates. These are research ethics trainings that are required for faculty doing research in the United States and are certified by the US Health and Human Services, Office of Science and Research.

**Table 3:** Learners in the Research Registry (by Admission Year)

Program	Cohorts Consented (Admission Year)	Total Number Approached	Number Consented	Consent Rate (of approached)
Medical School	2012–2018	1598	**1438**	90%
GME Total (Residents & Fellows)	2007–2018	**2627**	**1988**	**76%**

### Store data in a protected location, with limited access

A high level of protection for this type of data is mandated by these regulations. Establishing data protection protocols that are in-line with appropriate regulatory authorities is essential. Storage of protected information on an appropriately secure server is crucial for privacy protection [19, 20]. The MER registry includes educational data protected under The Family Educational Rights and Privacy Act of 1974 (FERPA) [21–24].

### Develop a standardized processes for registry data requests

Registry leadership needs to establish a process for making decisions about requests for data. A simple, well designed data request form that assess the degree of fit to the goals and regulations of the registry serves as an efficient way to begin the approval process and also serves to communicate those goals and regulations to potentially interested researchers. The registry consent applies only to routinely collected educational information as part of the required curriculum. This can cause confusion since researchers are often seeking to evaluate the impact of a curricular intervention. We often need to clarify that an intervention that is not a routine part of the curriculum - not available to all learners - would not meet registry criteria. In the case of an experimental design where only some students have access to the intervention, investigators need to approach the IRB directly for review. Once it is established that a request is appropriate, there needs to be a transparent, documented decision-making process, and timely feedback given to requestors.

The DREAM data request process is standardized (Table 4, Fig. 2). Interested collaborators speak with registry representatives informally to review the scope of covered data and approval requirements. They then complete a data request survey, which is housed in REDCap, an IRB approved data program that collects essential study information (Table 4) [18]. PrMEIR’s internal team (authors SZ, LA, CG, JW, AK) reviews and discusses each proposal, follows-up to request required additional information, and provides a final decision. A registry data request is often an iterative, conversation, consultation and negotiation between the researcher and registry leadership. During the approval process, registry officials and applicants discuss study design, assessment strategy, and research questions. As noted earlier, this process helps develop applicants as education researchers, helps share information and knowledge about data, and serves to build collaboration among education researchers and synergies in education research projects. The decision of all registry requests is recorded in a request log available for audit by the IRB.

**Table 4:** Registry Data Request Form

Questions	Response Options
Demographics	
**Name of PI**	Text box
**PI Role/ Title**	
**PI Department**	
**PI Email**	
**Are you the PI?**	Y/N
**Are you (or the PI) already named as a Co-Investigator in either the Medical Student Registry or the Resident Registry IRB?**	□ Co-Investigator in the MEDICAL STUDENT Registry
	□ Co-Investigator in the RESIDENT Registry
	□ None of the above
	□ Not sure
Research Study Details	
**Please list all relevant collaborators:**	Text box
**Please describe your proposed study’s RESEARCH QUESTION.**	
**Please indicate which of the following groups are included in your proposed study’s SAMPLE:**	□ Medical Students
	□ Residents
	□ Fellows
	□ Other
**Please describe your SAMPLE in greater detail (e.g., Class year or cohort, etc.).**	Text box
**Please indicate which of the following routinely collected educational data you would like to include in your proposed study:**	□ Knowledge exams
	□ Peer assessments
	□ OSCE performance
	□ Assessments of clinical performance
	□ Shelf Exams
	□ Step Exams
	□ Board and/or In-Service Exams
	□ 360 Assessments
	□ EHR/EMR (including chart reviews)
	□ Panel performance data
	□ Pre- and post-curriculum questionnaire data
	□ Program evaluation/QI data
	□ Needs assessment surveys/questionnaires
	□ Admissions/entrance data
	□ OTHER
**Please describe the data sets in greater detail and/or specify which OTHER data you are interested in.**	Text box
**When do you plan on using this data for your study?**	
**Please describe the general research design you are using in this proposed study.**	
Confirmation of Eligibility for Registry	
**Does this study involve ONLY routinely collected educational data?**	Y/N
**Does this study involve ONLY routinely collected educational data?**	
**Does this study introduce any new curricular activities or interventions that are being conducted SOLELY for the purpose of research?**	
**Does this study involve collecting new or additional data from learners SOLELY for the purpose of research?**	
**Is the delivery or the content of educational materials and/or experiences being affected by the proposed research study?**	
**Are you able to obtain the routinely collected educational data for your study?**	
**Do the routinely collected educational data elements include the learners’ names or other identifier (e.g. Kerberos ID)?**	
**How does the proposed study seek to contribute to improvements in medical education?**	Text box
**Any additional questions or concerns you would like to share?**	
Mandatory Documents	
**Please attach a copy of your current CV/Resume.**	File upload
**Please attach a copy of your current CITI Training Completion Report.**	

### Maintain transparency around data usage & research

There are many concerns around use of education data for research. Some believe that learners are becoming more reluctant to allow their educational data to be used for research [13]. In our experience, curricular leaders often argue that they have ownership over the data produced during the conduct of formal curriculum they direct. In the era of “big data”, the public has had reason to become increasingly concerned about data use and sharing happening without their knowledge or consent. Public surveys show large variation in trust over use of personal data. Concerns have heightened in the wake of recent privacy breaches on social media platforms and in workplace information systems [25, 26]. Medical Education researchers along with partners in the research regulation community must consciously and constantly monitor the protection of rights of research subjects. Pragmatically, this means that data usage plans should be outlined in detail, both verbally and in writing, throughout the research process and researchers must plan to communicate timely results to collaborators and stakeholders.

DREAM documentation outlines data use terms during consenting periods and shares updates with the PrMEIR/ ROMEO community regularly through annual email reminders. Registry personnel provide documentation of the terms of DREAM data usage as part of research training workshops and master’s programs for our community. The registry adheres to FERPA mandates by seeking explicit, plain-language consent to use learner data and by maintaining a record of all uses of those data. This process routinely evolves with the changing needs of registry users and within the context of the global data community. We continue to uphold that each learner’s data belongs to them as individuals and therefore they must give explicit consent for the collection, pooling, and analysis of their data.

### Engage in continuous quality improvement

Internal quality improvement processes are vital to making a MER registry a dynamic engine within a vibrant and productive MER community of practice rather than simply a bureaucratic burden. The review process must fall in line with standard IRB continuing review requirements, including updating enrollment numbers, adding and deleting team members, and approving updated consent forms and procedures. However, we recommend going beyond this and ensuring that critical team members discuss, document, and troubleshoot any issues that have developed during the previous year of implementation in light of the goals of and vision for the registry. The PrMEIR team meets regularly to discuss research, consenting of trainees, and dissemination updates as well as routinely reviews the goals for DREAM.

## Principle 3: a MER registry should add value to your institution or collaboration by creating a community of practice

### Ensure the registry is trustworthy

Trust is established by maintaining the integrity of the process and is vital in moments where risk or uncertainty exists [27]. In instances where trust is threatened, registry representatives must work to maintain and re-establish lost trust through regular, clear communication and transparency.

DREAM personnel work to be transparent about data collection, management, sharing and usage during initial consenting of subjects. Personnel maintain transparency after work is shared publicly (i.e. presentation and publication). We regularly respond to informational requests, make formal and informal presentations about the registry, and have basic information on our work publicly available on our website.

Over our 12-year history with consenting trainees, there has been just one, single complaint from a consenting subject about the registry raised to the IRB. This triggered a detailed audit requiring a full investigation of registry practices and review of data. Responding to this audit, which was time consuming and challenging at the time, required us to further strengthen our policies and practices and correct a few clerical mistakes (e.g. we used out-of-date IRB approved consent forms for a consenting session) unrelated to the complaint. In retrospect, this was a critically important and highly valuable “stress test” for the registry, providing much needed pressure to maintain rigorous trustworthy processes.

### Provide consultative services for approved registry researchers

To maximize the validity of DREAM based research and in the spirit of collaboration, PrMEIR and the larger ROMEO community offers mentorship, introduction to institutional resources (e.g. research librarians, database selection and use, consultative statisticians) and support (e.g. research assistance, transcription) pragmatically [28]. During the data request review process, study protocols and data analysis plans are refined. Registry researchers are routinely welcomed to registry management meetings, and to present at larger PrMEIR meetings for guidance and mentorship [28]. We have found that this enables researchers to hone their plans by refining research questions, identifying study appropriate conceptual and theoretical models, and crafting data analysis plans. When studies are underway, we provide assistance in abstract and manuscript preparation individually and in groups. Most commonly we find ourselves helping researchers put their work into the context of the existing literature and with articulating the implications of their research. Over time, this has allowed us to build a robust medical education research community in our institution.

### Create a space for a community of practice

Communities of practice are groups of people who come together physically and/ or virtually to collaboratively learn and share in developing a joint enterprise through establishing an engaging social structure and sharing expertise and resources [29]. The ROMEO unit began in 2005, having originally been designed as an interprofessional, interdepartmental collaborative community aimed at conducting medical education scholarship to improve patient and population health. With weekly research meetings, ROMEO served to build a community of researchers with shared passions for contributing to the MER field. The output of this community reflects a wide range of research areas and a rich network of collaboration (Table 2).

The DREAM registries are key projects that tie the ROMEO community into a rich network of collaborators across health professions and clinical disciplines, including psychology, learning and library sciences, informatics and more. Further insight into the history can be seen when looking at how data collection and structuring evolved to meet new demands, all of this, in summation, created a vibrant community of practice.

## Conclusions

MER registries have the potential to provide the much-needed infrastructure enabling the community to conduct programs of research that fill critical gaps in our understanding of the direct link between health professions education and the health of patients. This work has the potential to be transformational. We have built a community of practice within the NYU community through the building of DREAM and hope, by persisting in this work the full potential of this tool and the community will be realized. At present, researchers with access to the registry have focused primarily on curricular and program evaluation, learner competency assessment, and measure validation (Table 1). We hope to expand the output of the registry to include patient outcomes by linking learner educational and clinical performance across the UME-GME continuum and into independent practice. Future publications will reflect our efforts in reaching this goal and will highlight the long-term impact of our collaborative work.

We are also working toward making these de-identified education research data sets publicly available and in particular are working with other medical schools to implement similar registries in the hopes of being able to make comparable data from multiple institutions in order to explore the many contextual factors that are of interest to the medical education community. The advice outlined above, reflects our hard-earned learning about how to build and maintain a productive MER registry.

## Data Availability

Data sharing is not applicable to this article as no datasets were generated or analyzed during the current study. While data for DREAM participants is not currently publicly available, the authors are working to make them available in the future. In the meantime, the authors are collaborating in building additional  research registries within external medical education communities.

## Abbreviations

BHPr: Bureau of Health Professions
CK: Clinical Knowledge
CITI: Collaborative Institutional Training Initiative
DREAM: Database for Research on Academic Medicine
DREAM ID: DREAM Identification Number
FERPA: The Family Educational Rights and Privacy Act of 1974
FTE: Full-time Equivalent
GPA: Grade Point Average
GME: Graduate Medical Education
HIPAA: Health Insurance Portability and Accountability Act of 1996
HRSA: Human Resource Services Administration
IT: Information Technology
IRB: Institutional Review Board
MCIT: Medical Center Information Technology
MCAT: Medical College Admission Test
MER: Medical Education Research
NYU: New York University
OSCEs: Objective Structured Clinical Examinations
PI: Principal Investigator
PINDAR: Principal Investigator Development and Resources
PrMEIR: Program for Medical Innovations and Research
ROMEO: Research on Medical Education Outcomes
UME: Undergraduate Medical Education

## References

[1] Chahine S, Kulasegaram KM, Wright S, Monteiro S, Grierson LE, Barber C (2018). A call to investigate the relationship between education and health outcomes using big data. Acad Med.

[2] Triola MM, Campion N, McGee JB, et al. An XML standard for virtual patients: exchanging case-based simulations in medical education. AMIA Annu Symp Proc. Bethesda. 2007;2007:741–745. Published 2007 Oct 11. PMC2655833 18693935

[3] Sarpel U, Hopkins M, More F, Yavner S, Pusic M, Nick M (2013). Medical students as human subjects in educational research. Med Educ Online.

[4] Cook DA, Andriole DA, Durning SJ, Roberts NK, Triola MM (2010). Longitudinal research databases in medical education: facilitating the study of educational outcomes over time and across institutions. Acad Med.

[5] Arts DGT, de Keiser NF, Scheffer G-J (2002). Defining and improving data quality in medical registries: a literature review, case study, and generic framework. J Am Med Inform Assoc.

[6] Irgens L (2012). The origin of registry-based medical research and care. Acta Neurol Scand Suppl.

[7] Gonnella JS, Hojat M, Veloski JJ (2005). Abstracts: Jefferson longitudinal study of medical education, [full volume]. Jefferson Longitudinal Study of Medical Education.

[8] Gillespie C, Zabar S, Altshuler L, Fox J, Pusic M, Xu J (2016). The research on medical education outcomes (ROMEO) registry: addressing ethical and practical challenges of using “bigger,” longitudinal educational data. Acad Med.

[9] Jongbloed B, Enders J, Salerno C (2008). Higher education and its communities: interconnections, interdependencies and a research agenda. High Educ.

[10] Ellaway RH, Pusic MV, Galbraith RM, Cameron T (2014). Developing the role of big data and analytics in health professional education. Med Teacher.

[11] Kimball R, Reeves L, Ross M, Thornthwaite W (1998). The data warehouse lifecycle toolkit: expert methods for designing, developing, and deploying data warehouses: John Wiley & Sons.

[12] Solomon DJ, Henry RC, Hogan JG, Van Amburg GH, Taylor J (1991). Evaluation and implementation of public health registries. Public Health Rep.

[13] Ellaway RH, Topps D, Pusic M. Data, Big and Small: Emerging Challenges to Medical Education Scholarship. Acad Med. 2019;94(1):31-36. 10.1097/ACM.0000000000002465. 10.1097/ACM.0000000000002465 30256249

[14] Choi HJ, Lee MJ, Choi C-M, Lee J, Shin S-Y, Lyu Y (2015). Establishing the role of honest broker: bridging the gap between protecting personal health data and clinical research efficiency. PeerJ..

[15] El Emam K, Rodgers S, Malin B (2015). Anonymising and sharing individual patient data. Bmj.

[16] Dhir R, Patel AA, Winters S, Bisceglia M, Swanson D, Aamodt R (2008). A multidisciplinary approach to honest broker services for tissue banks and clinical data: a pragmatic and practical model. Cancer.

[17] Dokholyan RS, Muhlbaier LH, Falletta JM, Jacobs JP, Shahian D, Haan CK (2009). Regulatory and ethical considerations for linking clinical and administrative databases. Am Heart J.

[18] Harris PA, Taylor R, Thielke R, Payne J, Gonzalez N, Conde JG (2009). Research electronic data capture (REDCap)--a metadata-driven methodology and workflow process for providing translational research informatics support. J Biomed Inform.

[19] Mercuri RT (2004). The HIPAA-potamus in health care data security. Commun ACM.

[20] Choi YB, Capitan KE, Krause JS, Streeper MM (2006). Challenges associated with privacy in health care industry: implementation of HIPAA and the security rules. J Med Syst.

[21] Armstrong D, Kline-Rogers E, Jani SM, Goldman EB, Fang J, Mukherjee D (2005). Potential impact of the HIPAA privacy rule on data collection in a registry of patients with acute coronary syndrome. Arch Intern Med.

[22] O'Donnell ML (2002). FERPA: Only a piece of the privacy puzzle. JC & UL.

[23] Rinehart-Thompson LA (2009). Amendments to FERPA regulations: new changes attempt to balance safety and privacy in student records. J AHIMA.

[24] Weinberger JA, Michael JA (1976). Federal restrictions on educational research. Educ Res.

[25] Liao S (2018). New survey finds Americans’ trust in Facebook continues to decline The Verge.

[26] Thielsch MT, Meeßen SM, Hertel G (2018). Trust and distrust in information systems at the workplace. PeerJ.

[27] Mayer RC, Davis JH, Schoorman FD (1995). An integrative model of organizational trust. Acad Manag Rev.

[28] Sambunjak D, Straus SE, Marusic A (2010). A systematic review of qualitative research on the meaning and characteristics of mentoring in academic medicine. J Gen Intern Med.

[29] Wenger E (2011). Communities of practice: a brief introduction.

